# High‐dimensional Spatial Immune Profiling Highlights Microglia‐Like Cells in Human Dorsal Root Ganglia

**DOI:** 10.1002/eji.70211

**Published:** 2026-05-19

**Authors:** Marius Schwabenland, Busranur Oeztuerk, Thomas Blank, Juergen Beck, Bertram Bengsch, Marco Prinz

**Affiliations:** ^1^ Institute of Neuropathology, Faculty of Medicine University of Freiburg Freiburg Germany; ^2^ Department of Neurosurgery University Medical Center Freiburg Freiburg Germany; ^3^ Clinic for Internal Medicine II, Gastroenterology, Hepatology, Endocrinology, and Infectious Disease, Faculty of Medicine University Medical Center Freiburg Freiburg Germany; ^4^ Signalling Research Centres BIOSS and CIBSS University of Freiburg Freiburg Germany; ^5^ Center Brain Research and Advancements In Neuroimmunology (BRAIN), Faculty of Medicine University of Freiburg Freiburg Germany

**Keywords:** dorsal root ganglia, imaging mass cytometry, microglia, microglia‐like cells, myeloid cells, spatial immune profiling

## Abstract

The human dorsal root ganglia (DRG) are increasingly recognized as immunologically active sites within the peripheral nervous system. While single‐cell transcriptomics has recently identified myeloid populations with microglia‐like profiles in DRG across species, an in‐depth, spatially resolved protein‐level characterization in human tissue is lacking. Here, we used highly multiplexed Imaging Mass Cytometry (IMC) to map and phenotype myeloid cells in human DRG at subcellular resolution. A 41‐marker panel enabled in‐depth profiling of immune and neural cell types in situ. We identified a subset of Iba1^+^ myeloid cells co‐expressing canonical microglial markers such as P2RY12, TMEM119, and SLC2A5, located in close spatial proximity to neuronal somata. Unsupervised clustering (FlowSOM) of >6000 Iba1^+^ cells identified eight distinct clusters. Among them, distinct myeloid cell subsets exhibited a clear microglial‐like signature and were localized near neurofilament^+^ neurons. In contrast, Iba1^+^ clusters expressing CD68, HLA‐DR, and other activation markers were spatially segregated. These findings provide a spatially resolved, protein‑level atlas of Iba1^+^ myeloid subsets in human DRG and offer a resource for dissecting neuroimmune niches relevant to chronic pain and peripheral neuropathies.

## Graphical abstract

High‐dimensional imaging mass cytometry reveals diverse macrophage populations in human dorsal root ganglia (DRG). Microglia‐like cells, characterized by markers such as P2RY12, SLC2A5, and TMEM119, localize near neuronal structures, whereas other myeloid subsets are found in interstitial regions. Spatial analysis reveals distinct localization patterns within the DRG.

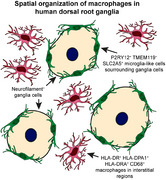

## 1 Introduction

Peripheral nerves and their associated ganglia are increasingly recognized as active immunological sites, particularly in the context of chronic pain, neuroinflammation, and neuropathies [1, 2]. The dorsal root ganglia (DRG) are known to host a diverse repertoire of immune cells, yet their phenotype, spatial organization, and function in human tissue remain poorly understood [3].

In rodent models, macrophage populations have been identified in the DRG under both homeostatic and pathological conditions, where they are visualized by expression of markers such as Iba1 [4, 5]. These resident macrophages appear to be a stable component of the sensory nervous system and participate in maintaining tissue integrity [4].

Peripheral nervous system (PNS) macrophages, including those residing in DRG, have been shown to represent distinct immune populations that expand upon injury and exhibit transcriptional features partially overlapping with central microglia [6, 7]. This underscores the immunological complexity of sensory ganglia and supports the view that DRG‐resident macrophages constitute a specialized arm of PNS immunity [6]. In the context of peripheral nerve injury, DRG macrophages have emerged as critical mediators of neuropathic pain [8]. Their depletion attenuated mechanical hypersensitivity, highlighting their essential role in the initiation and maintenance of pain signaling [8]. In another study, macrophages in the DRG were shown to support the enhanced regenerative capacity of sensory neurons after injury through neuron–macrophage interactions and the secretion of pro‑regenerative mediators such as oncomodulin [9].

A recent report highlights that DRG macrophages are not merely passive responders [10]. Using lineage tracing and single‐cell RNA sequencing, subsets of these cells were shown to be capable of self‐renewal and transition into a repair‐associated phenotype after injury, supporting axonal regeneration and modulating Schwann cell activity [10]. These findings highlight their distinct functional identity within the peripheral sensory nervous system.

Despite these insights from animal models, our understanding of immune populations in human DRG remains fragmentary. Early immunohistochemical studies identified cells expressing MHC class II in sensory ganglia [11, 12]. More recently, a single‐cell transcriptomic atlas focused mainly on neuronal subtypes and provided only superficial annotation of immune cells without phenotypic depth or spatial resolution [13]. Expanding on this, a cross‐species transcriptomic study identified microglia‐like myeloid cells in both rodent and primate DRG, including humans [14]. These cells expressed canonical microglial genes such as *P2RY12* and *TMEM119*, suggesting conserved microglial‐like features in peripheral ganglia across evolution [14, 15]. While immunohistochemistry confirmed P2RY12 protein expression in human DRG, the analysis was limited in scope and did not capture broader myeloid phenotypes or spatial distribution. Microglial cells of the central nervous system (CNS) are considered the main local immune cells of the brain parenchyma [16, 17, 18]. To what extent DRG macrophages share microglial features is still not completely clear.

These limitations underscore the need for a comprehensive, spatially resolved characterization of DRG immune populations at the protein level. To address this, we employed highly multiplexed imaging mass cytometry (IMC). This cytometry‐by‐time‐of‐flight (CyTOF)‐based platform enables the simultaneous detection of more than 40 protein markers at subcellular resolution in intact tissue, allowing precise mapping of immune and neural cell types in their native anatomical context. By integrating the high‐dimensional protein profiling data with spatial information, our study aimed to define the heterogeneity, tissue localization, and activation states of DRG myeloid cells, with special focus on those with microglial‐like signatures. This approach delivers an in‐depth characterization of myeloid cell diversity and distribution in human DRG, with potential implications for our understanding of pain mechanisms and peripheral neuropathies.

## 2 Results

### 2.1 Identification of Microglia‐Like Cells Using High‐Dimensional Imaging Mass Cytometry in Human Dorsal Root Ganglia

**FIGURE 1:**
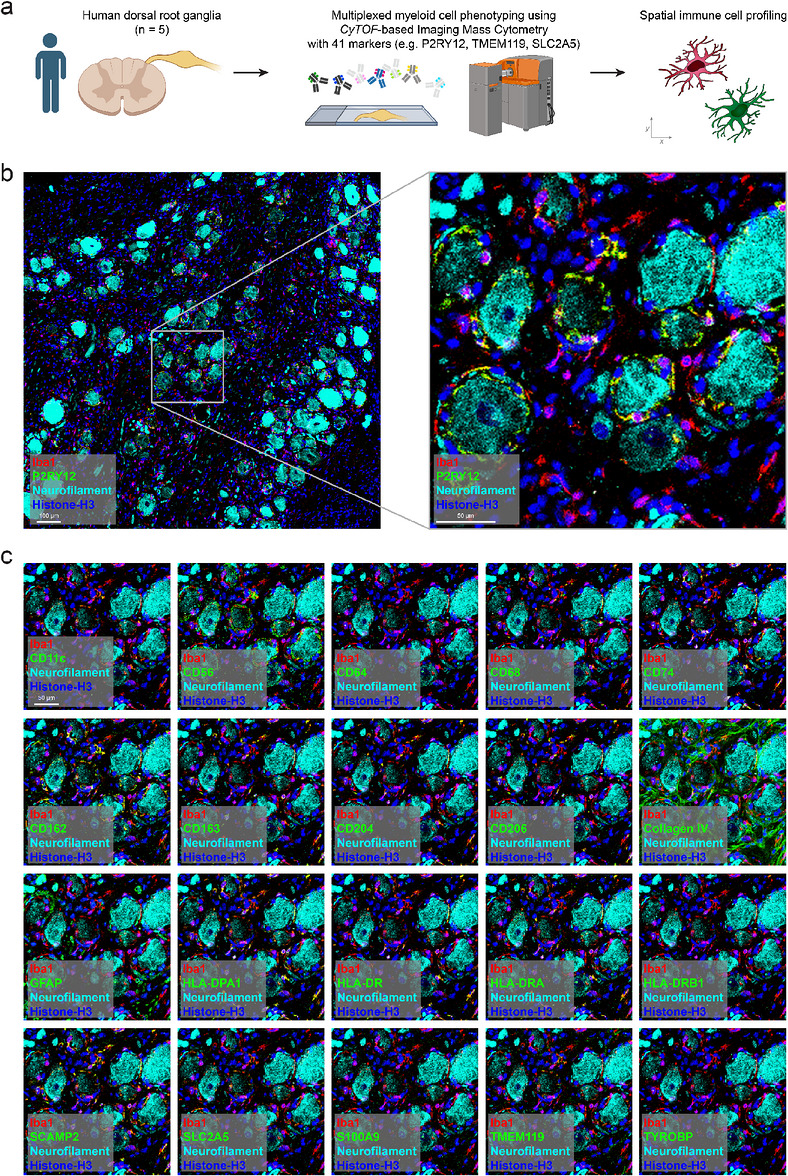
High‐dimensional imaging mass cytometry (IMC) identifies myeloid cells expressing microglial markers at protein level in healthy human dorsal root ganglia. (a) Experimental workflow. Tissue slices from healthy human dorsal root ganglia (*n* = 5 ganglia; 2 male and 3 female donors, aged 49–63 years) were analyzed by comprehensive spatial neuropathological analyses at the protein level using high‐dimensional cytometry‐by‐time‐of‐flight‐based imaging mass cytometry (IMC). CyTOF: Cytometry by time of flight. (b) Left panel: Representative images displaying imaging mass cytometry of human dorsal root ganglia. Iba1 is shown in red, P2RY12 in green, neurofilament in turquoise, and histone‐H3 in blue. Scale bar: 100 µm. Right panel: Zoomed‐in visualization of the sample referring to the left panel. Scale bar: 50 µm. (c) Visualization of different markers in the same area presented in (b). The respective marker indicated in the panel is shown in green, Iba1 in red, neurofilament in turquoise, and Histone‐H3 in blue. Scale bar: 50 µm.

To achieve a high‐resolution spatial and phenotypic characterization of immune cells in human dorsal root ganglia (DRG), we designed a 41‐marker IMC panel (Figure 1a). The panel was constructed to allow for the identification of key anatomical components of the DRG, including ganglion cells (Table 1).

**TABLE 1:** Imaging mass cytometry panel.

Channel	Epitope	Manufacturer	Catalog number	Clone	Stock concentration	Dilution	Working concentration
89 Y	CD45	Atlas	AMAb90518	CL0159	0.2 mg/mL	1:100	2 µg/mL
113 In	CD20	BD	555677	H1	0.2 mg/mL	1:50	4 µg/mL
115 In	HLA‐DR	abcam	ab176408	TAL 1B5	0.2 mg/mL	1:400	0.5 µg/mL
141 Pr	MBP	abcam	ab230378	EPR21188	0.2 mg/mL	1:100	2 µg/mL
142 Nd	Nestin	ProteinTech	29285‐1‐AP	polyclonal	0.2 mg/mL	1:100	2 µg/mL
143 Nd	GFAP	abcam	ab218309	EPR1034Y	0.2 mg/mL	1:200	1 µg/mL
144 Nd	INPP5D	Santa Cruz	sc‐8425	P1C1	0.2 mg/mL	1:100	2 µg/mL
145 Nd	Olig2	R&D	AF2418	polyclonal	0.2 mg/mL	1:100	2 µg/mL
146 Nd	TYROBP	Atlas	HPA041899	polyclonal	0.2 mg/mL	1:400	0.5 µg/mL
147 Sm	CD163	Novus	NB110‐40686	EDHU‐1	0.2 mg/mL	1:400	0.5 µg/mL
148 Nd	NeuN	Biolegend	834501	1B7	0.2 mg/mL	1:400	0.5 µg/mL
149 Sm	SMA	abcam	ab220795	EPR5368	0.2 mg/mL	1:1600	0.125 µg/mL
150 Nd	CD56	Biolegend	318345	HCD56	0.2 mg/mL	1:200	1 µg/mL
151 Eu	CD31	Fluidigm	3151025D	EPR3094	0.2 mg/mL	1:400	0.5 µg/mL
152 Sm	Ki‐67	Invitrogen	14‐5698‐82	SolA15	0.2 mg/mL	1:800	0.25 µg/mL
153 Eu	Neurofilament‐H	Biolegend	801701	SMI‐32P	0.2 mg/mL	1:200	1 µg/mL
154 Sm	Map2	DAKO	M4403	HM‐2	0.2 mg/mL	1:200	1 µg/mL
155 Gd	P2RY12	Atlas	HPA014518	polyclonal	0.2 mg/mL	1:800	0.25 µg/mL
156 Gd	CD4	Abcam	ab181724	EPR6855	0.2 mg/mL	1:200	1 µg/mL
157 Gd	CD64	Sino Biological	50086‐R008	008	0.2 mg/mL	1:100	2 µg/mL
158 Gd	MX1	abcam	ab284604	EPR24485‐19	0.2 mg/mL	1:400	0.5 µg/mL
159 Tb	CD68	Biolegend	916104	KP1	0.2 mg/mL	1:1600	0.125 µg/mL
160 Gd	HLA‐DRA	ProteinTech	17221‐1‐AP	polyclonal	0.2 mg/mL	1:200	1 µg/mL
161 Dy	Iba1	abcam	ab220815	EPR16588	0.2 mg/mL	1:1600	0.125 µg/mL
162 Dy	CD8a	Biolegend	372902	C8/144B	0.2 mg/mL	1:800	0.25 µg/mL
163 Dy	TMEM119	Sigma	AMab91528	CL8714	0.2 mg/mL	1:800	0.25 µg/mL
164 Dy	HLA‐DRB1	ProteinTech	15862‐1‐AP	polyclonal	0.2 mg/mL	1:100	2 µg/mL
165 Ho	HLA‐DPA1	ProteinTech	16109‐1‐AP	polyclonal	0.2 mg/mL	1:800	0.25 µg/mL
166 Er	CD204	invitrogen	14‐9054‐82	J5HTR3	0.2 mg/mL	1:800	0.25 µg/mL
167 Er	CD11c	Proteintech	60258‐1‐Ig	2F1C10	0.2 mg/mL	1:200	1 µg/mL
169 Tm	SLC2A5	Atlas	HPA005449	polyclonal	0.2 mg/mL	1:800	0.25 µg/mL
170 Er	CD3	CST	85061S	D7A6E	0.2 mg/mL	1:800	0.25 µg/mL
171 Yb	SCAMP2	Atlas	HPA014699	polyclonal	0.2 mg/mL	1:800	0.25 µg/mL
172 Yb	Collagen IV	Millipore	AB769	polyclonal	0.2 mg/mL	1:1600	0.125 µg/mL
173 Yb	CD162	Biolegend	328802	KPL‐1	0.2 mg/mL	1:400	0.5 µg/mL
174 Yb	CD74	Biolegend	326802	LN2	0.2 mg/mL	1:200	1 µg/mL
175 Lu	CD206	Atlas	AMAb90746	CL0387	0.2 mg/mL	1:400	0.5 µg/mL
176 Yb	S100A9	Atlas	AMAb91690	CL11191	0.2 mg/mL	1:1600	0.125 µg/mL
191 Ir	Iridium	Fluidigm	201192A	—	125 µM	1:500	0.25 µM
193 Ir	Iridium	Fluidigm	201192A	—	125 µM	1:500	0.25 µM
194 Pt	HH3	CST	4499BF	D1H2	0.2 mg/mL	1:100	2 µg/mL

*Note*: The table displays the details of the panel used for CyTOF‐based imaging mass cytometry (IMC).

Neuronal structures were visualized using neurofilament, neuronal nuclei antigen (NeuN), CD56 (neural cell adhesion molecule, NCAM1), and microtubule‐associated protein 2 (MAP2). To map tissue architecture and vascular compartments, we included collagen IV, CD31 (platelet endothelial cell adhesion molecule, PECAM1) for endothelial cells, and smooth muscle actin (SMA) for arterial smooth muscle cells.

To identify myeloid cells, the panel incorporated the pan‐myeloid marker ionized calcium‐binding adapter molecule 1 (Iba1). We included canonical microglial signature proteins such as purinergic receptor P2Y12 (P2RY12), transmembrane protein 119 (TMEM119), and solute carrier family 2 member 5 (SLC2A5). Those markers are typically expressed by homeostatic microglia in the central nervous system [15]. The panel further comprised markers typically expressed by CNS‐associated macrophages, such as perivascular, meningeal, or choroid plexus macrophages. These included CD163 (scavenger receptor cysteine‐rich type 1 protein M130), CD204 (macrophage scavenger receptor 1), and CD206 (mannose receptor C‐type 1). Several major histocompatibility complex (MHC) class II proteins, HLA‐DR, HLA‐DRA, HLA‐DRB1, and HLA‐DPA1, were added to assess antigen‐presenting capacity [19]. Phagocytic activity in myeloid cells was assessed using the lysosomal marker CD68 (macrosialin). Additional proteins used to characterize the myeloid compartment included secretory carrier membrane protein 2 (SCAMP2), a vesicle trafficking protein implicated in immune regulation, the adhesion molecule CD162 (P‐selectin glycoprotein ligand‐1), CD74 (HLA‐DR antigens‐associated invariant chain) which stabilizes MHC class II molecules and modulates macrophage activation, and CD64 (Fc gamma receptor I), a high‐affinity IgG receptor that mediates Fc‐dependent signaling and immune complex recognition in macrophages. We also incorporated S100 calcium‐binding protein A9 (S100A9) to detect monocytes [20]. To capture cells of the adaptive immune system, we used CD3, CD4, and CD8 for T‐cell subsets, and CD20 to identify B cells.

The panel was applied to five healthy human DRG samples. Neurofilament^+^, large ganglion cells were clearly identifiable based on their characteristic morphology and size (Figure 1b). Robust expression of Iba1 was detected in numerous ramified cells throughout the ganglia. These Iba1^+^ myeloid cells were observed surrounding neuronal somata and also located within the interstitial spaces between ganglion cells. Notably, expression of purinergic receptor P2Y12 (P2RY12) was detected in a subset of these Iba1^+^ cells in proximity to the ganglion cells, suggesting a spatially restricted microglia‐like phenotype.

Several additional myeloid markers exhibited spatial overlap with Iba1^+^ cells, consistent with their presence in the myeloid compartment (Figure 1c). However, their expression was restricted to subsets of Iba1^+^ cells. For instance, HLA‐DR and CD68 were predominantly found in Iba1^+^ cells within the interstitial regions between ganglion cells, but showed low or undetectable expression in P2RY12^+^ Iba1^+^ cells in proximity to neuronal somata. In contrast, CD162 was frequently expressed in Iba1^+^ cells located adjacent to ganglion cells, while only rarely detected in interstitial regions.

### 2.2 Unsupervised Profiling of Segmented Iba1^+^ Cells Reveals Phenotypic Diversity Across the Myeloid Compartment

**FIGURE 2:**
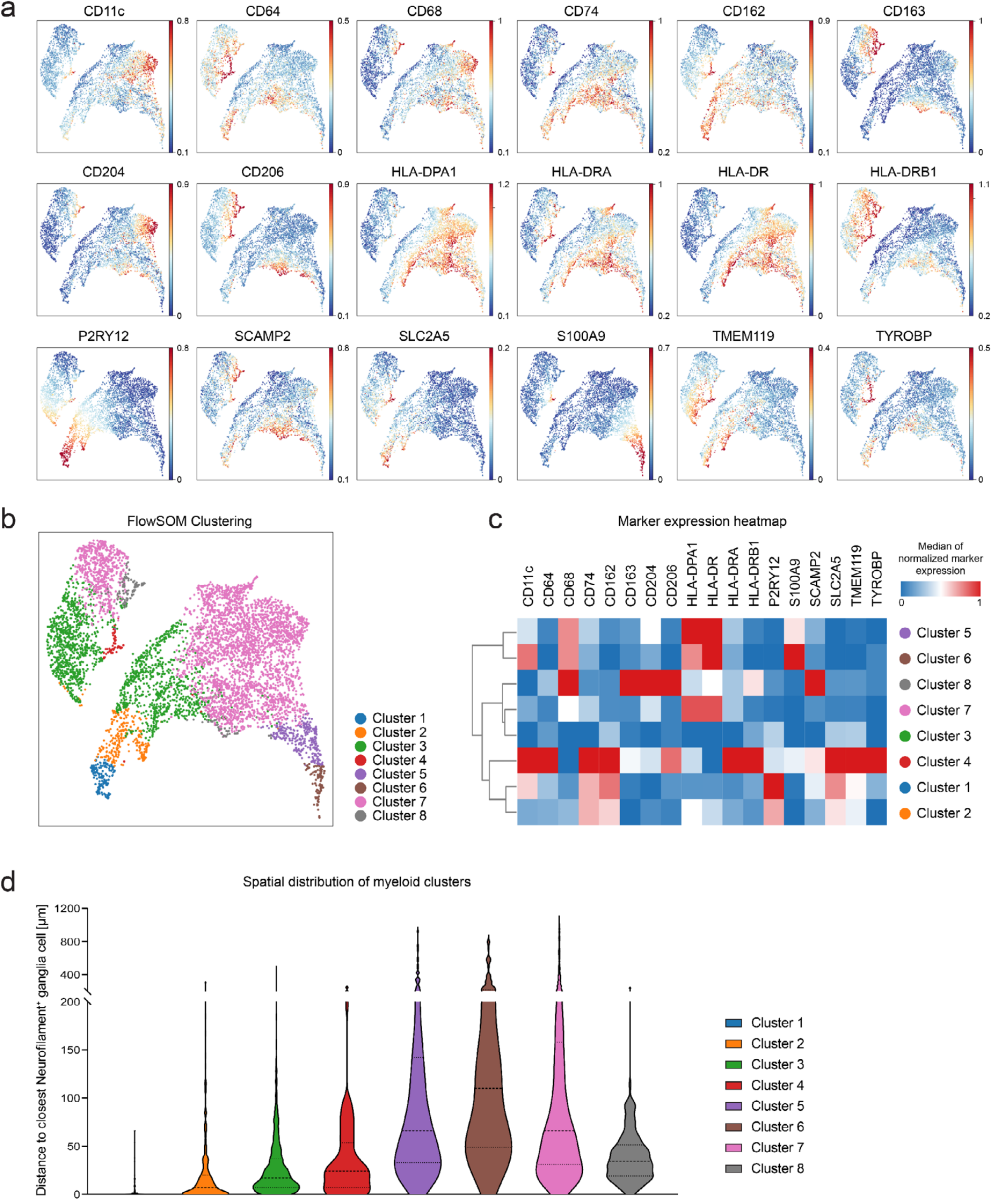
Identification and spatial localization of distinct myeloid clusters in human dorsal root ganglia. (a) Uniform manifold approximation and projection for dimension reduction (UMAP) plots of 6261 Iba1^+^ myeloid cells in human dorsal root ganglia. Single‐cell data were obtained from imaging mass cytometry after cell segmentation. One dot represents one cell. The normalized expression intensity of the respective marker is indicated by color. (b) UMAP plot of the same 6,261 Iba1^+^ myeloid cells shown in (a), now colored by cluster identity. Clustering was performed using FlowSOM, identifying eight distinct myeloid cell clusters, each represented by a different color. (c) Heatmap showing the median marker expression per cluster for the eight FlowSOM‐defined cell clusters shown in (b). Rows represent clusters, and columns represent markers. Color intensity reflects min‐max‐scaled normalized expression values, ranging from low (blue) to high (red). Clusters were grouped by similarity in marker expression using hierarchical clustering. (d) Violin plots showing the spatial distribution of each myeloid cluster in relation to the nearest Neurofilament^+^ ganglia cell. Values represent the distance in micrometers. Black dashed lines indicate the median; dotted lines denote the interquartile range (25th–75th percentile). Clusters are color‐coded as in (b, c).

To further characterize the myeloid cell compartment in human DRG, we segmented the IMC dataset and obtained protein expression profiles of single cells (Figure S1). Dimensionality reduction using Uniform Manifold Approximation and Projection (UMAP) was applied to the expression profiles of 6261 Iba1^+^ cells. The analysis uncovered a broad spectrum of marker expression patterns within the Iba1^+^ compartment, reflecting underlying cellular heterogeneity. Normalized expression intensities of selected markers are visualized in Figure 2a. To identify phenotypically distinct populations within this compartment, we performed unsupervised clustering using FlowSOM, which is based on a self‐organizing map approach, followed by aggregation to define eight distinct myeloid populations (Figure 2b; Figure S2). A heatmap displaying the median marker expression across these clusters is shown in Figure 2c, highlighting both shared and divergent phenotypic signatures among the identified subsets. Notably, cluster 1 exhibited the highest levels of P2RY12 as well as strong expression of the additional microglial core markers TMEM119 and SLC2A5. CD163, CD204, and CD206 showed minimal expression in this cluster. CD68 was only present at low levels, and no induction of MHC class II molecules, such as HLA‐DPA1, HLA‐DR, and HLA‐DRA, was observed. Clusters 2, 3, and 4 also expressed microglial core markers such as P2RY12, TMEM119, and SLC2A5. By contrast, clusters 5, 6, 7, and 8 lacked expression of microglial core markers and instead showed strong induction of MHC class II molecules. Clusters 5, 6, and 8 expressed high levels of CD68, indicating strong lysosomal activity. Cluster 7 showed moderate CD68 expression. Cluster 8 expressed CD68 together with CD163, CD204, and CD206. Cluster 6 expressed S100A9 and was identified as a monocyte‐enriched myeloid subset.

To investigate whether the identified myeloid clusters exhibited distinct anatomical localizations within the DRG, we analyzed the spatial distribution of each cluster in relation to the nearest neurofilament^+^ ganglion cell (Figure 2d). Clusters expressing microglial core markers (clusters 1–4) were located in proximity to ganglion cells, suggesting a specialized niche adjacent to neuronal somata. Clusters lacking microglial core marker expression were located at a greater distance from ganglia cells. These clusters were characterized by elevated expression of antigen‐presentation markers, including HLA‐DPA1, HLA‐DR, and HLA‐DRA (clusters 5–7). Cluster 8, which also lacked microglial core marker expression but instead exhibited CD163, CD204, and CD206 expression, was likewise predominantly located in interstitial regions. These spatial patterns support the presence of functionally and anatomically specialized myeloid cell populations within the human DRG.

To further characterize the spatial organization of myeloid cells within the DRG, we performed spatial neighborhood analysis using lisaClust. This approach identified eight distinct macrophage neighborhoods based on the local spatial arrangement of FlowSOM‐defined myeloid populations (Figure S3a). Mapping these neighborhoods to the identified myeloid clusters revealed distinct distribution patterns, indicating that specific macrophage populations preferentially occupy defined spatial niches within the tissue (Figure S3b). In addition, these neighborhoods differed in their distance to the nearest neurofilament‐positive ganglion cell, supporting the presence of anatomically distinct microenvironments within the DRG (Figure S3c). These findings are consistent with the spatial patterns observed at the single‐cluster level and further support the existence of functionally specialized macrophage populations in the human DRG.

## 3 Discussion

Peripheral sensory ganglia, including the dorsal root ganglia, are increasingly appreciated as immunologically active sites that host myeloid cell populations. While transcriptomic studies have recently identified microglia‐like populations in human DRG, protein‐level data capturing their phenotypic diversity and anatomical context have been lacking. Our study addresses this gap by applying high‐dimensional IMC to human DRG, enabling multiplexed immune phenotyping within intact tissue architecture.

By combining a 41‑marker panel with unsupervised clustering of more than 6000 Iba1^+^ cells, we generated a detailed spatial atlas of myeloid cells within the human DRG. This analysis revealed substantial heterogeneity, identifying eight discrete clusters with distinct marker profiles and anatomical niches. Among these, four clusters emerged as a microglia‐like subset characterized by expression of P2RY12, TMEM119, and SLC2A5, but minimal expression of lysosomal CD68 and MHC class II markers. This phenotype, together with its preferential localization adjacent to neurofilament^+^ ganglion cells, suggests a specialized, likely homeostatic role reminiscent of parenchymal microglia in the central nervous system.

In contrast, clusters such as 5, 6, and 7 displayed strong induction of MHC class II molecules (HLA‑DRA, HLA‑DRB1, and HLA‑DPA1), consistent with enhanced antigen‐presenting capacity. These clusters also expressed high levels of CD68, indicating increased lysosomal activity. Additional subsets (Cluster 8) expressed markers such as CD163, CD204, and CD206, aligning with phenotypes described for perivascular macrophages. Cluster 6, defined by strong S100A9 expression, was identified as a monocyte‐enriched subset.

Our spatial analysis revealed that myeloid subsets occupy distinct tissue niches within the DRG. Microglia‑like cells were concentrated near neuronal somata, consistent with neuron‑supportive or homeostatic functions. In contrast, clusters with strong antigen‑presentation and lysosomal signatures were dispersed throughout the interstitial space, where they may serve as immune sentinels or first responders to peripheral signals. This alignment of phenotype with anatomical location points to a compartmentalized organization of myeloid functions within the DRG.

Our findings expand on previous transcriptomic studies that identified DRG myeloid populations with microglial signatures but did not resolve their spatial context or broader phenotypic diversity [14]. By incorporating a wide array of activation, antigen‑presentation, and functional markers, our IMC approach reveals a continuum of states ranging from homeostatic, P2RY12^+^ TMEM119^+^ SLC2A5^+^ microglia‑like cells near neurons to more activated, antigen‑presenting subsets in interstitial regions. These phenotypic and spatial insights provide new granularity on how DRG‑resident macrophages may contribute to neuronal maintenance, immune surveillance, and potentially to pain mechanisms or neuropathic priming, aligning with prior observations of their roles in regeneration and tissue homeostasis.

Altogether, our study delivers the first spatially resolved, high‑dimensional protein atlas of myeloid cells in the human DRG. By integrating phenotypic profiling with anatomical context, we provide a resource for future investigations into how distinct myeloid niches shape sensory neuron function and contribute to pain mechanisms, regeneration, and peripheral neuropathies.

## 4 Data Limitations and Perspectives

Our study is limited by the number of analyzed human DRG samples and the cross‐sectional nature of the dataset. While our multiplexed IMC panel enabled comprehensive profiling of the myeloid compartment, it cannot resolve dynamic or functional responses under pathological conditions. Future studies incorporating disease cohorts and integrative transcriptomic and proteomic approaches will be instrumental in elucidating the role of microglia‐like cells in chronic pain and neuropathic disease.

## 5 Materials and Methods

### 5.1 Imaging Mass Cytometry

Imaging Mass Cytometry was following adapted protocols from previous studies [21, 22]. Briefly, antibodies were metal‐conjugated using the Maxpar X8 labeling kit (Standard BioTools). Tissue sections (4 µm) from formalin‐fixed paraffin‐embedded (FFPE) healthy dorsal root ganglia (*n* = 5 ganglia; 2 male and 3 female donors, aged 49–63 years) were deparaffinized and subjected to heat‐induced epitope retrieval for 40 min using EnVision FLEX Target Retrieval Solution, High pH (DAKO, cat. #K8000). Following a blocking step with SuperBlock (ThermoFisher, cat. #37581), sections were incubated overnight at room temperature with the antibody cocktail (Table 1) diluted in 0.5% BSA and 1% Triton X‐100 in TRIS buffer. Nuclear staining was performed using the Iridium Cell‐ID Intercalator (Standard BioTools, cat. #201192A) for 30 min. IMC data acquisition was carried out using the Hyperion Imaging System (Fluidigm/Standard BioTools). Image segmentation was conducted using the IMCSegmentationPipeline as described previously [23].

### 5.2 Image Visualization and Data Analysis

Processed images were visualized using MCD Viewer (v1.0.560.6). For single‐cell analysis, Iba1^+^ myeloid cells were gated based on a minimum expression threshold of ≥2 and a segmented cell area of ≥ 20 µm^2^.

To investigate phenotypic diversity within the Iba1^+^ myeloid compartment, we applied UMAP using the cloud‐based cytometry analysis platform omiq.ai. Single‐cell data were obtained from segmented Iba1^+^ cells, with expression values normalized to Iba1 intensity and arcsinh‐transformed. Dimensionality reduction was performed using 18 phenotypic and functional markers, selected to reflect myeloid identity, activation, and immune regulation: CD11c, CD64, CD68, CD74, CD162, CD163, CD204, CD206, HLA‐DPA1, HLA‐DR, HLA‐DRA, HLA‐DRB1, P2RY12, S100A9, SCAMP2, SLC2A5, TMEM119, and TYROBP. UMAP was run with 15 nearest neighbors and a minimum distance of 0.4, using Euclidean distance, spectral initialization, and a learning rate of 1 over 200 training epochs.

Unsupervised clustering of Iba1^+^ single‐cell expression profiles was performed using the FlowSOM algorithm to identify phenotypically distinct myeloid subsets. The analysis was based on the same set of 18 markers used for UMAP projection. A 7 × 7 self‐organizing map was trained for 20 iterations using Euclidean distance, followed by consensus metaclustering to define eight final clusters.

To visualize phenotypic diversity across myeloid clusters, we generated a clustered heatmap of median marker expression per FlowSOM‐defined cluster (Figure 2c). Expression values were Min‐Max normalized per marker to a scale from 0 (minimum) to 1 (maximum) to enable comparability across features. Hierarchical clustering of clusters was performed using Euclidean distance and Ward linkage, with dendrograms displayed for rows. Markers were displayed in alphabetical order.

Violin plots were generated using GraphPad Prism version 9.5.1. Each violin displays the distribution of distances to the nearest neurofilament^+^ ganglion cell per cluster. Medians are represented by black dashed lines, and interquartile ranges (25th–75th percentile) by black dotted lines.

Spatial neighborhood analysis was performed using the lisaClust package. Briefly, local indicators of spatial association (LISA) were computed based on the spatial arrangement of FlowSOM‐defined myeloid populations. LISA curves were evaluated across multiple spatial scales (radii of 10, 20, and 50 µm), capturing local cellular composition within increasing neighborhood sizes. The resulting LISA profiles were clustered using k‐means clustering (*k* = 8), yielding eight distinct macrophage neighborhoods.

## Author Contributions

M.S. and B.O. performed experiments and analyzed the results. M.P. supervised the project. M.P. and M.S. wrote the manuscript. T.B., J.B., and B.B. were involved in designing parts of the project and contributed to the writing of the manuscript.

## Ethics Statement

This study was conducted in accordance with the principles expressed in the Declaration of Helsinki and its amendments. Ethical approval was obtained from the Ethics Committee of the University of Freiburg (211/20 and 10008/09).

## Patient Consent Statement

All human tissue samples were collected in compliance with institutional and national regulations. Procedures were approved by the Ethics Committee of the University of Freiburg.

## Conflicts of Interest

The authors declare no conflicts of interest.

## Supporting information




**Supporting File**: eji70211‐sup‐0001‐SupMat.pdf.

## Data Availability

All data supporting the findings of this study have been deposited in a publicly available repository and can be accessed at https://doi.org/10.5281/zenodo.19299768.
