# Supplementary material for: High‐dimensional Spatial Immune Profiling Highlights Microglia‐Like Cells in Human Dorsal Root Ganglia
Source: Eur J Immunol. 2026 May 19;56:e70211. doi: 10.1002/eji.70211 (PMC13185684; doi:10.1002/eji.70211)
Supplement: Supplementary file 1 — Supporting File: eji70211‐sup‐0001‐SupMat.pdf. [file EJI-56-e70211-s001.pdf]

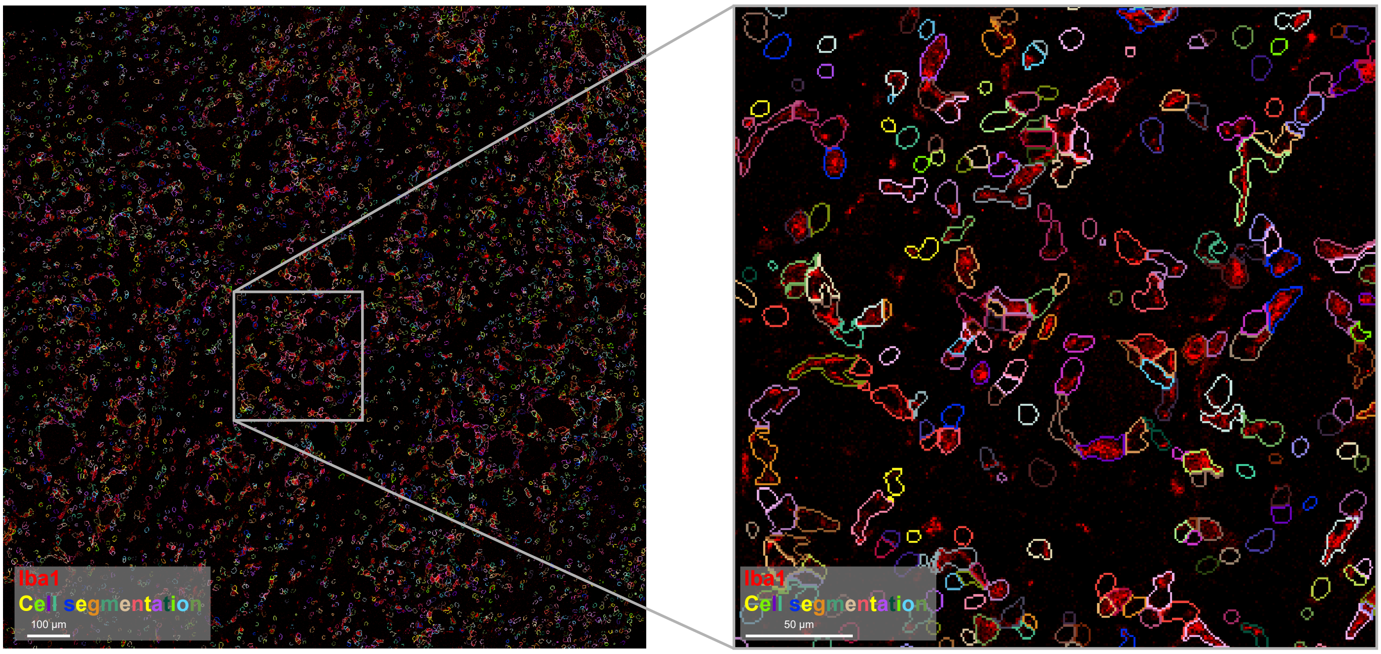

Supplementary Figure 1: Visualization of cell segmentation used for single-cell feature extraction. Representative IMC image showing Iba1 signal (red) with overlaid segmentation boundaries generated during image processing. Segmentation contours corresponding to individual cells are displayed in different colors to aid visual separation of neighboring cells. The right panel shows a magnified region highlighting individual segmented cells. Scale bars: 100 µm (left) and 50 µm (right).

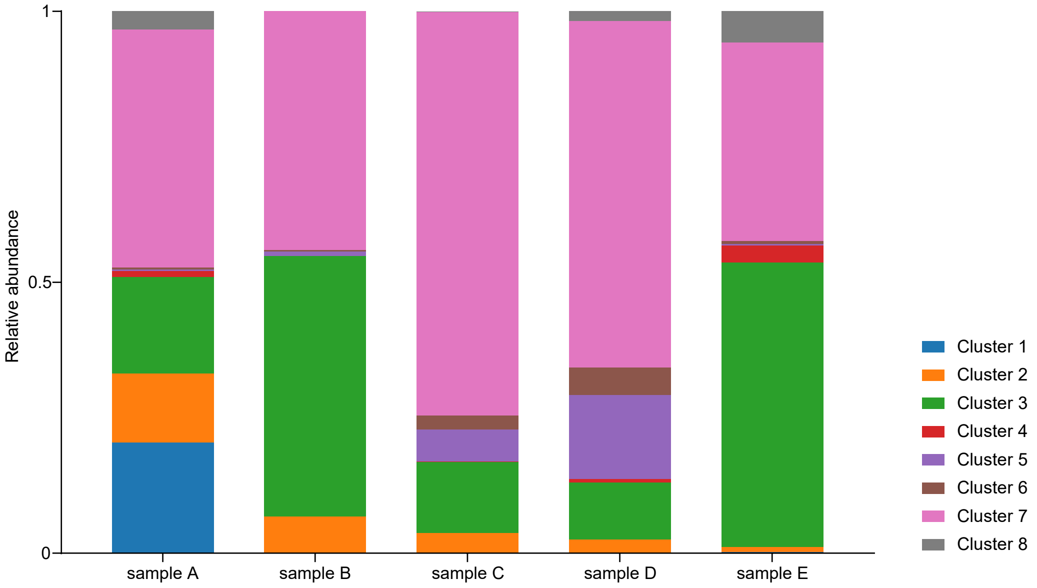

Supplementary Figure 2. Relative abundance of macrophage populations across individual samples. Stacked bar plot showing the relative abundance of FlowSOM-defined macrophage populations in each individual DRG sample. Each bar represents one sample, and colors indicate the proportional contribution of each macrophage cluster.

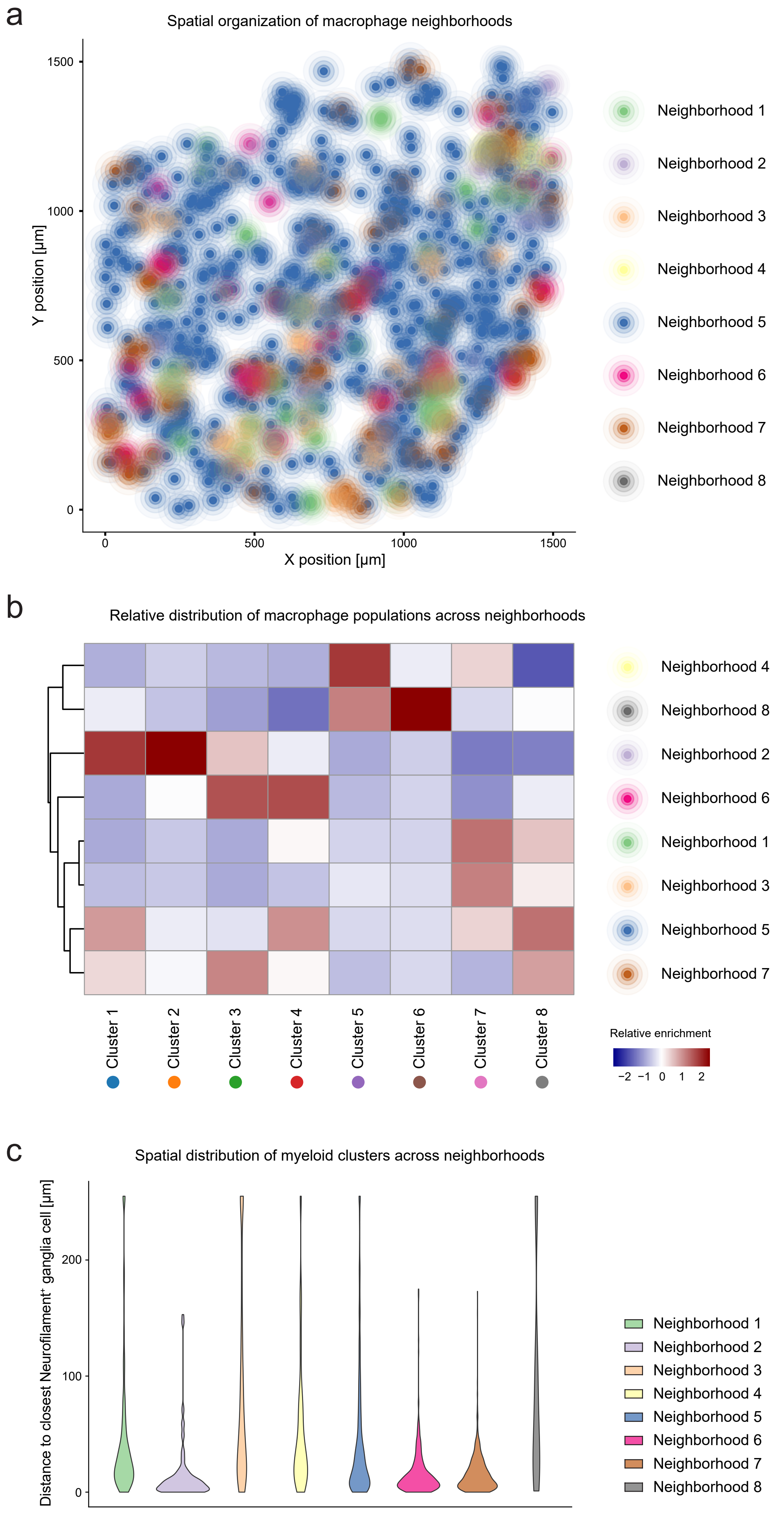

Supplementary Fig. 3

Supplementary Figure 3: Spatial organization of macrophage neighborhoods in human DRG.
(a) Representative DRG sample showing spatial neighborhoods identified by lisaClust. Neighborhoods were defined based on local cellular organization and grouped into eight distinct classes. Each point represents one Iba1$^+$ cell and is colored according to its assigned region.
(b) Heatmap showing the distribution of FlowSOM-defined macrophage populations across spatial neighborhoods identified by lisaClust. Rows represent spatial regions and columns represent FlowSOM clusters. Colors indicate the relative enrichment of each cluster across neighborhoods.
(c) Violin plots showing the distribution of distances of cells assigned to each lisaClust neighborhood to the nearest neurofilament-positive ganglion cell. This analysis illustrates that neighborhoods differ in their anatomical relationship to neuronal structures within the DRG.
